# Methylation of WNT target genes *AXIN2* and *DKK1* as robust biomarkers for recurrence prediction in stage II colon cancer

**DOI:** 10.1038/oncsis.2017.9

**Published:** 2017-04-03

**Authors:** R Kandimalla, J F Linnekamp, S van Hooff, A Castells, X Llor, M Andreu, R Jover, A Goel, J P Medema

**Affiliations:** 1 Laboratory for Experimental Oncology and Radiobiology (LEXOR), Center for Experimental Molecular Medicine (CEMM), Academic Medical Center (AMC), University of Amsterdam, Amsterdam, The Netherlands; 2 Cancer Genomics Center, Amsterdam, The Netherlands; 3 Center for Gastrointestinal Research and Center for Epigenetics, Cancer Prevention and Cancer Genomics, Baylor Scott and White Research Institute and Charles A Sammons Cancer Center, Baylor University Medical Center, Dallas, TX, USA; 4 Institut de Malaties Digestives i Metabòliques, CIBERehd, Hospital Clínic, Barcelona, Spain; 5 University of Yale, New Haven, CT, USA; 6 Gastroenterology Department, Hospital del Mar, Barcelona, Spain; 7 Servicio de Medicina Digestiva, Hospital General Universitario de Alicante, Instituto de Investigación Sanitaria ISABIAL, Alicante, Spain

## Abstract

Stage II colon cancer (CC) still remains a clinical challenge with patient stratification for adjuvant therapy (AT) largely relying on clinical parameters. Prognostic biomarkers are urgently needed for better stratification. Previously, we have shown that WNT target genes *AXIN2*, *DKK1*, *APCDD1*, *ASCL2* and *LGR5* are silenced by DNA methylation and could serve as prognostic markers in stage II CC patients using methylation-specific PCR. Here, we have extended our discovery cohort AMC90-AJCC-II (*N*=65) and methylation was analyzed by quantitative pyrosequencing. Subsequently, we validated the results in an independent EPICOLON1 CC cohort (*N*=79). Methylation of WNT target genes is negatively correlated to mRNA expression. A combination of *AXIN2* and *DKK1* methylation significantly predicted recurrences in univariate (area under the curve (AUC)=0.83, confidence interval (CI): 0.72–0.94, *P*<0.0001) analysis in stage II microsatellite stable (MSS) CC patients. This two marker combination showed an AUC of 0.80 (CI: 0.68–0.91, *P*<0.0001) in the EPICOLON1 validation cohort. Multivariate analysis in the Academic Medical Center (AMC) cohort revealed that both WNT target gene methylation and consensus molecular subtype 4 (CMS4) are significantly associated with poor recurrence-free survival (hazard ratio (HR)_methylation_: 3.84, 95% CI: 1.14–12.43; HR_CMS4_: 3.73, 95% CI: 1.22–11.48). CMS4 subtype tumors with WNT target methylation showed worse prognosis. Combining WNT target gene methylation and CMS4 subtype lead to an AUC of 0.89 (0.791–0.982, *P*<0.0001) for recurrence prediction. Notably, we observed that methylation of DKK1 is high in BRAF mutant and CIMP (CpG island methylator phenotype)-positive cancers, whereas AXIN2 methylation appears to be associated with CMS4. Methylation of *AXIN2* and *DKK1* were found to be robust markers for recurrence prediction in stage II MSS CC patients. Further validation of these findings in a randomized and prospective manner could pave a way to identify poor prognosis patients of stage II CC for AT.

## Introduction

Colorectal cancer (CRC) is a leading cause of cancer-related deaths worldwide. In 2016, there were estimated 95 270 newly diagnosed colon cancer (CC) cases, and 49 190 deaths in the United States.^1^ Survival of patients is closely related with the tumor stage at the time of diagnosis as 5-year relative survival rates range from 65% for all stages, 90% local, 71% regional and 13% in distant disease (SEER Cancer Statistics Review, 1975–2010; http://seer.cancer.gov/csr/1975_2010). Post-surgery, adjuvant therapy (AT) is only recommended to those with high-risk stage II, as well as stage III and IV tumors.^2^ However, the prognosis of low-risk stage II patients who have undergone curative surgery is relatively good as relapse occurs only in a small fraction of them.^3, 4^ Therefore, identifying these relapse-prone patients could significantly contribute to the optimization of treatment selection. Currently, the management of stage II CRC patients after curative surgery mainly depends on the presented histopathologic characteristics defined by the tumor size, number of lymph nodes investigated, differentiation, perforation, obstruction and lymphovasular invasion. Based on the presented clinical risk factors, patients are divided into low or high risk and the AT is advised in the latter case. However, this may lead to the under or over treatment of low- and high-risk groups. The association of CpG island methylator phenotype (CIMP), microsatellite instability (MSI) and mutations in BRAF and KRAS with the prognosis has been studied extensively and it has been found that MSI patients have a good prognosis and should not receive AT in stage II.^5, 6, 7, 8^ Therefore, there is an urgent need for molecular biomarkers for a better stratification of patients who will be benefitted by the adjuvant chemotherapy, especially in microsatellite stable (MSS) stage II CRC patients.

We previously identified that methylation of a set of five WNT target genes was associated with recurrence in a small set of 23 stage II CC patients, demonstrating their prognostic potential.^9^ In this study, we have extended our test set findings to 65 stage II MSS CC patients using highly sensitive pyrosequencing method and subsequently validated independently in a population-based EPICOLON1 cohort.

## Results

### Pyrosequencing assay for detecting WNT target gene methylation showed high concordance with MSP

**Figure 1:**
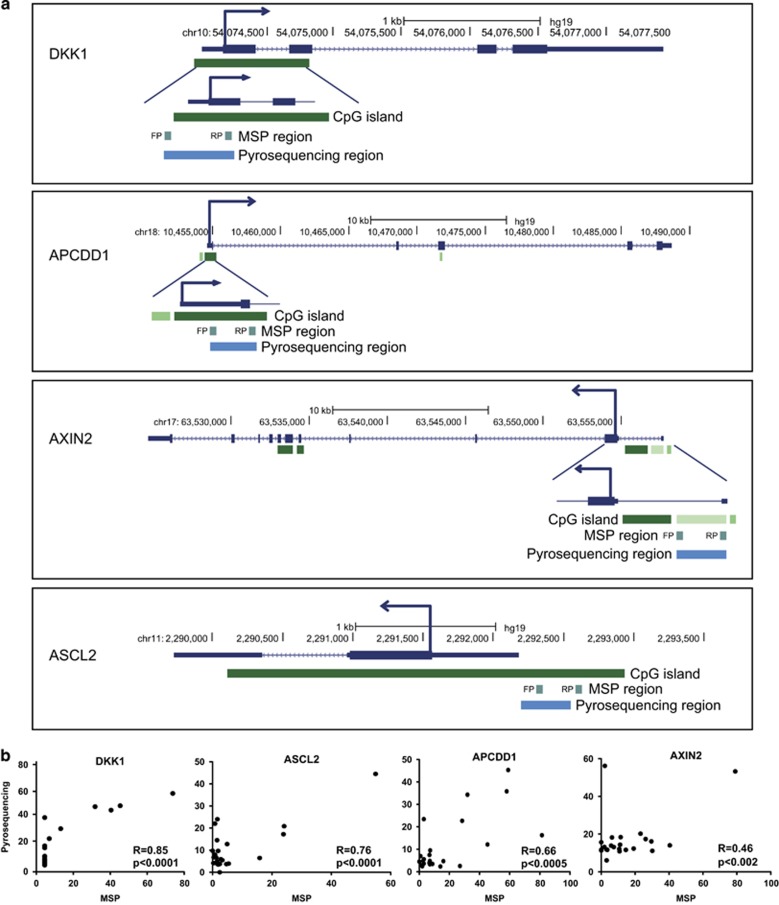
(**a**) An illustration of CpG regions of the four genes analyzed by MSP and pyrosequencing. (**b**) Correlation analyses of earlier MSP results to the pyrosequencing data of the 23 patients. FP, forward primer; MSP, methylation specific PCR; RP, reverse primer.

Methylation of *AXIN2, DKK1, APCDD1, ASCL2* and *LGR5* was determined by methylation-specific PCR (MSP) in our previous study.^9^ To be able to design a feasible diagnostic assay, we developed pyrosequencing assays for more sensitive and quantitative detection of methylation. As illustrated in Figure 1a, we designed pyrosequencing PCR primers corresponding to the region previously analyzed by MSP. Raw data of pyrosequencing are given in the Supplementary Table 1. The region, which is pyrosequenced, is within the promotor CpG island for all the five genes. Owing to a very low methylation level of *LGR5* in the extended test set, which is consistent with the earlier analysis using MSP, we therefore omitted this from any further analysis. The number of CpGs spanning in the pyrosequencing assay are 10 for *APCDD1*, *ASCL2* and 9 for *AXIN2*, *DKK1*. The concordance in methylation between the adjacent CpGs is very high for all the four genes and we used average methylation for our analysis (Supplementary Table 2). We have performed a correlation analysis on the MSP and pyrosequencing results of the 23 patients from our earlier study. As shown in Figure 1b, we found a very strong and significant correlation between the two methods as the correlation coefficients are 0.85, 0.76, 0.66 and 0.46 for DKK1, ASCL2, APCDD1 and AXIN2, respectively, with a *P*-value of <0.05 for all the four genes.

### WNT target gene methylation association with expression and other clinical parameters

To determine whether WNT target gene methylation is associated with expression and other clinical parameters, we performed Pearson correlation and Mann–Whitney *U-*test in the Academic Medical Center (AMC) test set. Methylation of *AXIN2*, *APCDD1* and *ASCL2* are negatively correlated significantly with gene expression in a Pearson correlation analysis. The *R*-value and *P*-values of Pearson correlation are as following: *AXIN2* (*R*=−0.27, *P*<0.03), *APCDD1* (*R*=−0.40, *P*<0.001) and *ASCL2* (*R*=−0.33, *P*<0.007). DKK1 showed a negative correlation but not significant (*R*=−0.20, *P*<0.113). The association of WNT target genes methylation with CIMP, mutations in *BRAF*, *P53*, *KRAS* and consensus molecular subtype 4 (CMS4) performed by Mann–Whitney *U*-test revealed a significant correlation of *DKK1* hypermethylation to CIMP (*P*<0.001) and *BRAF* (*P*<0.0001), whereas *AXIN2* hypermethylation to CMS4 subtype (*P*<0.017).

### Validation of WNT target gene methylation association with recurrence-free survival in an extended test set

**Figure 2:**
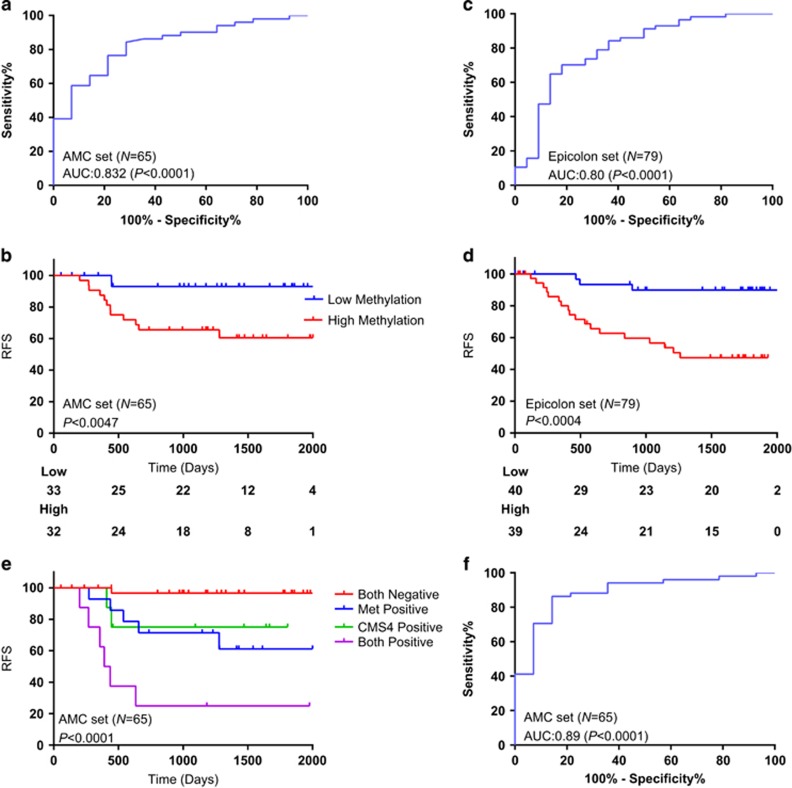
(**a**) Receiver operating characteristic (ROC) curve measuring the sensitivity and specificity of methylation assay (*DKK1*+*AXIN2*) in recurrence prediction within the AMC test set (*N*=65). (**b**) KM curve and log-rank analysis of the methylation assay dichotomized to low and high methylation groups in the AMC test set (*N*=65). (**c**) ROC curve measuring the sensitivity and specificity of methylation assay (*DKK1*+*AXIN2*) in recurrence prediction within the EPICOLON1 validation set (*N*=79). (**d**) KM curve and log-rank analysis of the methylation assay dichotomized to low and high methylation groups in the EPICOLON1 validation set (*N*=79). (**e**) KM curve and log-rank analysis of the methylation assay stratified on CMS4 subtype in predicting recurrence-free survival (AMC test set, *N*=65). (**f**) ROC curve measuring the sensitivity and specificity of methylation assay (*DKK1*+*AXIN2*) in combination with CMS4 for recurrence prediction within the AMC test set (*N*=65).

To extend our test set, we used all stage II MSS CC patients from the AMC90-AJCCII cohort in this study. We excluded the MSI patients owing to the hypermethylated phenotype and good prognosis compared with the MSS patients. We analyzed methylation of *AXIN2, DKK1, APCDD1* and *ASCL2* in 65 stage II MSS CRC patient cohort using pyrosequencing assays. In our earlier study, we used a rank-sum approach to combine the individual markers for recurrence prediction. Here, we performed Cox regression analysis using backward elimination method (Table 1). This analysis revealed a combination of *AXIN2*+*DKK1* as the best model for recurrence prediction. The model combining *AXIN2*+*DKK1* methylation resulted in an area under the curve (AUC) of 0.83 (confidence interval (CI): 0.72–0.94, *P*<0.0001) for recurrence prediction (Figure 2a). In the univariate analysis, along with WNT target gene methylation, *BRAF* mutation and CMS4 are found to be significant in recurrence prediction. A multivariate analysis revealed that WNT target gene methylation (HR_methylation_: 3.84, 95% CI: 1.14–12.43) and CMS4 subtype are (HR_CMS4_: 3.73, 95% CI: 1.22–11.48) independent predictors of recurrence. Table 2 displays the univariate and multivariate analysis of different variables for recurrence prediction. As depicted in Figure 2b, the Kaplan–Meier (KM) curve showed significant drop in recurrence-free survival in patients with high WNT target gene methylation (*P*<0.0047).

### Independent validation of WNT target gene methylation association with recurrence prediction in the EPICOLON1 cohort

To confirm our findings of test cohort, we next set out to perform independent validation in a similar patient cohort as the AMC test set. From the independent EPICOLON1 cohort, we selected stage II, MSS and untreated patients for the validation. The methylation percentages of *AXIN2* and *DKK1* are analyzed by pyrosequencing and the methylation percentages are depicted in Supplementary Table 1. We performed an independent Cox regression analysis on the combination of *AXIN2*+*DKK1* in the EPICOLON1 cohort. As in the test cohort, we used a median cutoff derived from the two gene Cox model. This resulted in an AUC of 0.80 (CI: 0.681–0.915, *P*<0.0001) for recurrence prediction (Figure 2c) in this independent validation cohort. As depicted in Figure 2d, KM analysis showed a significant decrease in the recurrence-free survival in patients with high WNT target gene methylation (*P*<0.0004).

### CMS4 subtype CC patients with high WNT target gene methylation show worse prognosis

The multivariate analysis performed on the AMC test set clearly indicated that the CMS4 subtype and WNT target gene methylation are independent predictors of recurrence. To obtain more insight into the clinical relevance of CMS4 and WNT target methylation, we performed the KM analysis on combination of CMS and WNT target gene methylation. As depicted in Figure 2e, patients who belong to CMS4 subtype with high WNT target gene methylation showed the worse prognosis. When we combined the CMS4 with WNT target gene methylation, we achieved an AUC of 0.887 (CI: 0.791–0.982, *P*<0.0001) for recurrence prediction (Figure 2f). Only one recurrence was missed out of 14 by combining CMS4 and WNT methylation. Six out of 14 recurrences were predicted by both CMS4 and WNT methylation, whereas two recurrences were predicted exclusively by CMS4 and five exclusively by WNT methylation. The interaction of WNT target gene methylation to CMS4 subtype is intriguing and the mechanism behind this need to be studied further.

## Discussion

Previously, we showed that a low WNT target gene expression as a result of promotor methylation was associated with poor prognosis in stage II CC.^10^ In this study, we have further strengthen this by extending the association with a bigger panel of untreated stage II MSS CC patients using a more sensitive pyrosequencing assay, and subsequently validated independently in a population-based EPICOLON1 patient cohort. The total number of patients analyzed in two cohorts is 144 and of these 36 patients had a recurrence. Methylation of WNT target genes *APCDD1, AXIN2, DKK1* and *ASCL2* are found to be reliable biomarkers for recurrence prediction, whereas the combination of *AXIN2* and *DKK1* was independently validated for recurrence prediction. *AXIN2*+*DKK1* methylation significantly predicted recurrence independent of age, sex, tumor size, localization, differentiation, CIMP, *BRAF* and *KRAS* mutations. The association of CIMP, MSI and mutations in *BRAF* and *KRAS* with prognosis has been studied extensively in earlier studies and generally MSI patients are shown to have good prognosis.^5, 6, 7, 8, 11, 12, 13, 14^ Many commercial prognostic assays based on gene expression profiling have been developed in the past few years. Namely, Oncotype DX Colon Cancer Assay, ColoPrint and OncoDefender–CRC.^15, 16, 17^ However, these methods are still not widely used in the clinical setting.

Recently, a gene expression-based consensus molecular clustering has been proposed, where CMS4 subtype CRC patients are found to be associated with poor prognosis^18^ and show low WNT activity. This low WNT activity may be due to the high WNT target gene methylation observed in this group of patients. Interestingly, we found that the CMS4 subtype patients with WNT target gene methylation have the worst recurrence-free survival. This apparent biological association needs further substantiation and investigation. Unlike CMS4 subtyping, which needs whole-genome expression profiling data, WNT target gene methylation is easy to perform and the reproducibility and sensitivity of the pyrosequencing assay is very high and can be adapted for a routine diagnostic assay in the future. WNT target gene methylation was better at recurrence prediction than the CMS classification in our test set. Addition of CMS4 to WNT target methylation assay resulted in a marginal increase in recurrence prediction. Owing to the lack of genetic and transcriptomic data for the EPICOLON1 validation cohort, we could not validate the association of WNT target genes with mutations, as well as CMS4 subtype.

*AXIN2*, *DKK1* and *APCDD1* are well-known negative feedback regulators of the WNT pathway^19^ and the epigenetic silencing of these genes may result in increased WNT activity and thereby tumorigenesis, progression and relapse. Although we found a significant negative correlation between WNT target gene methylation and mRNA expression, measuring methylation is more potent to gene expression. DNA methylation is a stable modification, which can be sensitively measured using pyrosequencing, where we sequence a region of 100–150 base pairs in assigning a patient in to low- or high-risk category. However, gene expression can be hampered by poor-quality RNA, as well as tumor cell purity, whereas immunohistochemistry can be challenging with the lack of sensitive antibodies, as well as interpersonal variation. In the previous study, we have also shown that treating the CRC cell lines with *DNMT1* inhibitor 5-azacytidine reduces proliferation *in vitro*. This may open up a new treatment strategy for CRC patients in future. We are currently working on understanding the association of methylation-mediated silencing by overexpressing *AXIN2* in CRC cell lines and xenografts. This will shed new light on this association in near future. Furthermore, we have found that *APC* mutations and *AXIN2* methylation are mutually exclusive in a panel of CRC cell lines, as well as in CRC TCGA cohort (data not shown). This may be of interest and needs to be further validated in other data sets. Many previous studies have found that WNT target genes are methylated in CRC and are potential diagnostic markers. Among those most validated diagnostic markers are *SFRP2*,^20^ *WNT2*^21^ and *WIF1*.^22^ A few other studies have also found methylation-based biomarkers for predicting progression in CRC, which includes *Myopodin*,^23^ *KISS1*,^24^ *hMLH1*,^25^ *HPP1* and *HLTF*.^26, 27, 28^ However, most studies lack independent validation. This emphasizes the potential of methylation-based biomarkers for diagnosis and prognosis of CRC and the need of independent validations, as well as large-scale prospective and randomized trials for the clinical translation.

One of the potential limitations of the prognostic markers is that it is not sufficient to only select patients for AT, but sensitive predictive markers are needed to select patients who will respond to the therapy. WNT methylation could potentially stratify patients for adjuvant chemotherapy using demethylating agents although this needs to be substantiated in pre-clinical settings.

In conclusion, WNT target gene methylation can serve as a robust biomarker for recurrence prediction in stage II CC. Extending these finding to other stages of CC may be an interesting next step. Further validation of these markers in multicenter prospective and randomized settings will help to translate these markers in to the clinic for the stratification of stage II CC patients into low- and high-risk groups.

## Materials and methods

### Patient cohorts

The test set cohort was obtained from the 65 AJCC stage II MSS CC patients who underwent surgical resection at the Amsterdam Medical Center, The Netherlands, in the years 1997–2006 (AMC-AJCCII-90). The study was approved by the medical ethical committee of the AMC. Informed consent was obtained from all subjects. For all these patients, extensive clinical and epidemiological data with a long-term clinical follow-up was available. Previously,^9^ we have performed gene expression profiling, MSI, CIMP, *BRAF*, *KRAS* and *TP53* analysis on these patients and these data were used to analyze the association of WNT target gene methylation with these variables. CMS subtyping information for the AMC-AJCCII90 cohort was obtained from Guinney *et al.*^18^ For independent validation of recurrence markers, we selected stage II MSS untreated CC patients from the population-based Spanish EPICOLON1 cohort (*N*=79). Patient demographics and clinical characteristics are provided in Table 3.

### DNA isolation, bisulfite conversion and pyrosequencing

For the test cohort, we used fresh frozen primary tissues for genomic DNA isolation and subsequently 500 ng of genomic DNA was used for bisulfite conversion using Epitect BS conversion kit from Qiagen (Hilden, Germany) according to the manufacturer’s instruction. Bisulfite conversion of EPICOLON1 FFPE cohort was performed by EZ-DNA methylation Gold-Kit (Zymo, Irvine, CA, USA). Methylation detection was carried out using the PyroMark PCR system (Qiagen). Bisulfite converted DNA was amplified using PyroMark PCR Mastermix from Qiagen using the recommended protocol. Primers were designed using the PyroMark Assay Design Software 2.0 (Qiagen) and span 250 base pairs including 10 CpGs in the *APCDD1* promoter region, 279 base pairs including 10 CpGs in the *ASCL2* promoter region, 229 base pairs including 9 CpGs in the *AXIN2* promoter region and 189 base pairs including 9 CpGs in the *DKK1* promoter region. PCR reactions and pyrosequencing were performed following the manufacturer’s instructions. Analysis is performed on the average methylation of all CpGs of a particular gene taken together. Primer sequences: *APCDD1*-forward: 5′-AGGTTTTAGAGTAGGATTGGAAATGT-3′; *APCDD1*-reverse: 5′-ACCCCCTCTCCCAAAACTA-3′ *APCDD1*-sequencing primer: 5′-AGTAGGATTGGAAATGTT-3′. *ASCL2*-forward:5′-GTTTGGAAGTTTAAGTTTATTAGT-3′; *ASCL2*-reverse: 5′-TTCCTCTACCTACACCTTCCTA-3′; *ASCL2*-sequencing primer: 5′-GGATTTGGGTAGTGTG-3′. *AXIN2*-forward: 5′-GTTTTTTTGGAGTTGATGGTAT-3′ *AXIN2*-reverse: 5′-AAATCTAAACTCCCTACACACTT-3′; *AXIN2*-sequencing primer: 5′-TTTGGAGTTGATGGTAT-3′. *DKK1*-forward: 5′-GGTTTTGTTGTTTTTTTTTTAAGG-3′ *DKK1*-reverse: 5′-CACTTTACAAACCTAAATCCC-3′ *DKK1*-sequencing primer: 5′-TTGTTGTTTTTTTTTTAAGG-3′.

### Statistical analysis

Receiver operating characteristic, survival, univariate and multivariate Cox regression analysis, Mann–Whitney *U*-test and Pearson correlation analysis was performed using IBM SPSS version 23 (Armonk, NY, USA) and R 3.2.4 (Vienna, Austria). Cox regression was performed on the four markers (*APCDD1, AXIN2, DKK1* and *ASCL2*) and the backward elimination method resulted in a model with two genes (*AXIN2* and *DKK1*) as best predictor (Table 1). Recurrence-free survival was measured from the day of surgery to the recurrence or the end of follow-up. The predictive probability values derived from the *AXIN2*+*DKK1* combination was used to plot the AUCs. To plot the KM curves, we dichotomized the patients based on a median cutoff chosen from the predictive probability values derived from Cox model (Supplementary Figure 1). Patients who are above median are considered highly methylated and below are considered lowly methylated. To analyze correlations between methylation and gene expression, we performed Pearson’s correlation coefficient in SPSS version 23. To analyze the association between Wnt target gene methylation and CIMP, *BRAF*, *KRAS, P53,* CMS4, we used Mann–Whitney *U*-test. Benjamini–Hochberg method was used to correct for multiple hypothesis testing wherever applicable. A *P*-value of below 0.05 was considered significant. For univariate and multivariate analysis, the following clinical and genetic information was used. Age, sex, T-stage, differentiation, location of the tumor, CIMP, *BRAF*, *KRAS*, *TP53*, CMS4 classification and methylation of two gene combination (dichotomized data). Only the significant variables in the univariate model were used to perform the multivariate analysis. We performed an independent Cox regression on EPICOLON1 cohort using *AXIN2* and *DKK1* methylation levels investigated by the pyrosequencing. The predictive probability values derived from the *AXIN2*+*DKK1* combination was used to plot the AUCs. To plot the KM curves, we dichotomized the patients using the median predictive probability value as a cutoff (Supplementary Figure 1). Patients who are above median are considered highly methylated and below are considered lowly methylated.

## Figures and Tables

**Table 1:** Cox regression analysis of *APCDD1*, *AXIN2*, *DKK1* and *ASCL2* in AMC cohort

	*B*	*s.e.*	*Wald*	*df*	*Sig.*	*Exp(B)*
*Step 1*						
APCDD1	0.019	0.036	0.265	1	0.607	1.019
AXIN2	0.133	0.047	7.881	1	0.005	1.142
DKK1	0.04	0.025	2.696	1	0.101	1.041
ASCL2	0.041	0.039	1.106	1	0.293	1.042
						
*Step 2*						
AXIN2	0.145	0.044	10.637	1	0.001	1.156
DKK1	0.047	0.02	5.557	1	0.018	1.048
ASCL2	0.049	0.035	1.934	1	0.164	1.051
						
*Step 3*						
**AXIN2**	**0.144**	**0.047**	**9.438**	**1**	**0.002**	**1.154**
**DKK1**	**0.042**	**0.019**	**4.872**	**1**	**0.027**	**1.043**

Abbreviation: AMC, Academic Medical Center. Bold entries represent the final significant model derived from cox regression backward elimination method.

**Table 2:** Univariate and multivariate analysis of recurrence-free survival using clinical, epidemiological and WNT target gene methylation in the AMC test set

	*Univariate*	*Multivariate*			
*Variable*	*Sig.*	*Sig.*	*HR*	*95.0% CI for Exp(B)*	
Age median	0.251				
Sex	0.478				
T4 vs T3	0.17				
Diff (good/mod vs poor)	0.669				
Location (left vs right)	0.557				
CIMP (pos vs neg)	0.877				
BRAF (Mut vs Wt)	**0.004**	0.686	2.410	0.634	9.153
KRAS (Mut vs Wt)	0.393				
P53 (Mut vs Wt)	0.278				
CMS4 vs rest	**0.001**	**0.021**	3.743	1.220	11.482
DKK1_AXIN2 (high vs low)	**0.0006**	**0.03**	3.841	1.141	12.934

Abbreviations: AMC, Academic Medical Center; CI, confidence interval; CIMP, CpG island methylator phenotype; CMS4, consensus molecular subtype 4; HR, hazard ratio. Bold entries represent the significant variables of univariate and multivariate models.

**Table 3:** Patient demographics and clinical characteristics

*Characteristics*	*AMC test set (*N*=65)*	*Epicolon validation set (*N*=79)*
*Age*		
Median	73	76
		
*Gender*		
Male	35	46
Female	30	30
NA		3
		
*Localization*		
Left	39	51
Right	26	25
NA		3
		
*Stage*		
II	65	79
		
*Grade*		
Well	1	14
Moderate	51	55
Poor	6	3
NA	7	7
		
*Relapse*		
Yes	14	22
No	51	57
		
*MSI*		
MSS	65	79
MSI		

Abbreviations: AMC, Academic Medical Center; MSI, microsatellite instability; MSS, microsatellite stable; NA, not applicable.
