# Supplementary material for: Methylation of WNT target genes AXIN2 and DKK1 as robust biomarkers for recurrence prediction in stage II colon cancer
Source: Oncogenesis. 2017 Apr 3;6(4):e308–. doi: 10.1038/oncsis.2017.9 (PMC5520503; doi:10.1038/oncsis.2017.9)
Supplement: Supplementary Table 1 [file oncsis20179x1.docx]

| **Supplementary Table 1: Wnt target gene methylation percentages in AMC65 test** | | | | |
| --- | --- | --- | --- | --- |
| **Sample** | **APCDD1_Methylation** | **AXIN2_Methylation** | **DKK1_Methylation** | **ASCL2_Methylation** |
| COL001 | 6.74 | 18.10 | 5.65 | 7.24 |
| COL002 | 2.04 | 11.60 | 2.65 | 4.34 |
| COL005 | 4.39 | 10.70 | 4.95 | 6.81 |
| COL006 | 4.18 | 15.00 | 33.14 | 4.65 |
| COL007 | 4.13 | 17.20 | 6.33 | 7.27 |
| COL010 | 5.66 | 14.50 | 8.86 | 8.88 |
| COL011 | 4.74 | 20.20 | 7.23 | 12.77 |
| COL014 | 4.22 | 10.80 | 5.22 | 8.68 |
| COL015 | 3.15 | 7.30 | 12.46 | 6.52 |
| COL017 | 3.59 | 11.60 | 5.01 | 8.43 |
| COL018 | 3.06 | 13.30 | 5.24 | 5.61 |
| COL019 | 4.82 | 10.90 | 7.01 | 14.53 |
| COL022 | 2.73 | 10.10 | 20.56 | 7.35 |
| COL023 | 2.98 | 11.20 | 4.76 | 8.65 |
| COL026 | 3.65 | 18.40 | 6.00 | 3.71 |
| COL033 | 4.14 | 14.90 | 9.54 | 7.17 |
| COL034 | 4.51 | 16.10 | 6.92 | 9.70 |
| COL036 | 27.00 | 24.40 | 26.60 | 7.48 |
| COL037 | 3.28 | 15.50 | 7.09 | 5.54 |
| COL038 | 2.56 | 6.10 | 2.98 | 24.03 |
| COL039 | 2.44 | 12.10 | 4.61 | 3.54 |
| COL041 | 2.09 | 8.10 | 3.19 | 2.93 |
| COL044 | 6.48 | 8.90 | 54.55 | 3.75 |
| COL045 | 3.47 | 17.90 | 4.91 | 5.85 |
| COL046 | 2.05 | 11.90 | 3.43 | 2.95 |
| COL047 | 3.39 | 12.20 | 4.28 | 4.05 |
| COL048 | 2.79 | 8.30 | 3.99 | 4.07 |
| COL049 | 2.36 | 15.70 | 4.00 | 9.73 |
| COL050 | 1.78 | 14.30 | 3.31 | 5.77 |
| COL051 | 3.09 | 11.70 | 6.90 | 4.45 |
| COL053 | 35.83 | 11.80 | 42.81 | 3.76 |
| COL054 | 22.69 | 14.00 | 8.58 | 44.52 |
| COL055 | 3.89 | 11.80 | 3.54 | 3.50 |
| COL056 | 6.94 | 11.30 | 3.97 | 4.18 |
| COL057 | 4.35 | 12.40 | 8.97 | 4.26 |
| COL059 | 31.49 | 18.60 | 12.85 | 6.20 |
| COL062 | 5.62 | 10.60 | 8.68 | 0.00 |
| COL063 | 3.06 | 10.50 | 7.69 | 5.60 |
| COL065 | 7.46 | 12.30 | 14.55 | 7.99 |
| COL066 | 3.76 | 13.10 | 3.74 | 6.97 |
| COL068 | 9.85 | 19.90 | 6.42 | 4.45 |
| COL069 | 3.66 | 11.70 | 20.57 | 0.00 |
| COL070 | 5.56 | 11.60 | 5.26 | 3.94 |
| COL072 | 5.56 | 17.20 | 3.51 | 1.98 |
| COL073 | 8.37 | 11.40 | 3.63 | 1.01 |
| COL074 | 4.08 | 14.90 | 5.81 | 4.09 |
| COL075 | 16.27 | 18.20 | 20.74 | 6.48 |
| COL076 | 3.30 | 12.50 | 4.15 | 6.32 |
| COL077 | 3.27 | 10.80 | 7.48 | 4.28 |
| COL078 | 9.54 | 11.10 | 8.96 | 6.73 |
| COL079 | 29.19 | 18.40 | 33.35 | 0.00 |
| COL080 | 3.75 | 17.60 | 5.29 | 6.72 |
| COL082 | 2.80 | 18.30 | 28.15 | 3.12 |
| COL083 | 4.24 | 16.80 | 5.50 | 8.55 |
| COL086 | 3.77 | 11.70 | 5.34 | 8.69 |
| COL089 | 4.88 | 11.70 | 6.71 | 5.49 |
| COL090 | 3.39 | 14.80 | 5.53 | 4.93 |
| COL092 | 12.13 | 56.20 | 15.87 | 0.00 |
| COL094 | 3.47 | 13.90 | 5.62 | 5.95 |
| COL095 | 3.92 | 13.20 | 34.88 | 4.89 |
| COL096 | 20.78 | 14.90 | 35.84 | 4.81 |
| COL097 | 2.67 | 13.70 | 3.41 | 6.58 |
| COL098 | 2.96 | 11.70 | 4.13 | 4.08 |
| COL101 | 3.26 | 17.50 | 5.57 | 9.32 |
| COL102 | 4.15 | 10.10 | 4.92 | 3.49 |

| **Supplementary Table 1: Wnt target gene methylation percentages in epicolon validation set** | | |
| --- | --- | --- |
| **Sample** | **AXIN2 methylation** | **DKK1 methylation** |
| 1001 | 20.8 | 0.4 |
| 1018 | 12.6 | 11.7 |
| 1023 | 11.6 | 3.98 |
| 1029 | 20.5 | 7.11 |
| 1034 | 8.7 | 2.56 |
| 1060 | 5.7 | 3.21 |
| 1061 | 12.0 | 0.0 |
| 1065 | 9.8 | 1.6 |
| 1074 | 16.5 | 0.0 |
| 1076 | 11.4 | 5.17 |
| 1081 | 6.2 | 2.71 |
| 1084 | 6.2 | 46.58 |
| 1092 | 8.0 | 6.72 |
| 1096 | 31.2 | 1.56 |
| 1097 | 37.9 | 9.16 |
| 1124 | 11.3 | 4.6 |
| 1135 | 8.7 | 9.91 |
| 1142 | 22.2 | 5.9 |
| 1145 | 6.4 | 5.24 |
| 1149 | 9.6 | 13.96 |
| 1150 | 12.9 | 7.9 |
| 1152 | 10.0 | 1.1 |
| 1162 | 12.5 | 2.7 |
| 1184 | 16.5 | 7.4 |
| 4018 | 14.6 | 0.6 |
| 4032 | 16.1 | 7.6 |
| 4045 | 13.3 | 1.1 |
| 6013 | 16.9 | 8.1 |
| 6074 | 7.1 | 1.6 |
| 6082 | 13.1 | 4.6 |
| 6104 | 8.2 | 3.2 |
| 7004 | 9.5 | 7.07 |
| 7030 | 15.6 | 0.1 |
| 7052 | 6.4 | 0.4 |
| 7067 | 8.7 | 5.3 |
| 7075 | 11.1 | 4.4 |
| 7084 | 11.3 | 14.8 |
| 7127 | 8.0 | 4.8 |
| 7130 | 3.4 | 0.0 |
| 7141 | 9.8 | 2.4 |
| 7148 | 11.8 | 3.0 |
| 7154 | 4.6 | 3.09 |
| 7162 | 13.2 | 3.3 |
| 7170 | 10.3 | 4.1 |
| 9001 | 12.0 | 11.0 |
| 9003 | 5.2 | 1.3 |
| 9004 | 9.8 | 17.8 |
| 9012 | 7.1 | 0.4 |
| 9027 | 8.1 | 32.0 |
| 9039 | 16.6 | 4.46 |
| 9052 | 7.6 | 4.75 |
| 9059 | 17.1 | 1.4 |
| 9071 | 2.6 | 3.1 |
| 9072 | 7.3 | 3.0 |
| 9081 | 9.0 | 1.6 |
| 9085 | 9.1 | 0.3 |
| 12001 | 3.3 | 0.6 |
| 12017 | 12.7 | 15.1 |
| 12035 | 11.8 | 0.7 |
| 12091 | 16.4 | 3.18 |
| 12115 | 9.6 | 5.1 |
| 15122 | 6.5 | 3.3 |
| 15123 | 4.5 | 4.59 |
| 26015 | 11.6 | 0.9 |
| 26023 | 5.8 | 1.6 |
| 26026 | 17.2 | 5.62 |
| 26052 | 5.2 | 0.7 |
| 26063 | 13.8 | 3.7 |
| 26068 | 14.8 | 0.5 |
| 28008 | 5.9 | 10.36 |
| 28037 | 7.0 | 1.4 |
| 28038 | 17.4 | 2.2 |
| 28039 | 23.9 | 0.7 |
| 28041 | 16.8 | 4.52 |
| 28044 | 2.8 | 4.43 |
| 28049 | 11.1 | 1.5 |
| 28080 | 5.2 | 1.9 |
| 28088 | 16.9 | 2.6 |
| 28092 | 9.5 | 4.45 |
