# Supplementary material for: Methylation of WNT target genes AXIN2 and DKK1 as robust biomarkers for recurrence prediction in stage II colon cancer
Source: Oncogenesis. 2017 Apr 3;6(4):e308–. doi: 10.1038/oncsis.2017.9 (PMC5520503; doi:10.1038/oncsis.2017.9)
Supplement: Supplementary Table 2 [file oncsis20179x2.docx]

**Supplementary Table 2:** Correlation analysis of methylation levels between adjacent CpGs in *APCDD1*, *AXIN2*, *DKK1* and *ASCL2* measured by pyrosequencing in the AMC test set
