# Supplementary material for: Methylation of WNT target genes AXIN2 and DKK1 as robust biomarkers for recurrence prediction in stage II colon cancer
Source: Oncogenesis. 2017 Apr 3;6(4):e308–. doi: 10.1038/oncsis.2017.9 (PMC5520503; doi:10.1038/oncsis.2017.9)

Supplementary Figure 1: Median cut-off chosen from the predictive probability value of two gene cox regression model
A) AMC test set B) Epicolon validation set (NR-No recurrence, R-Recurrence)

A

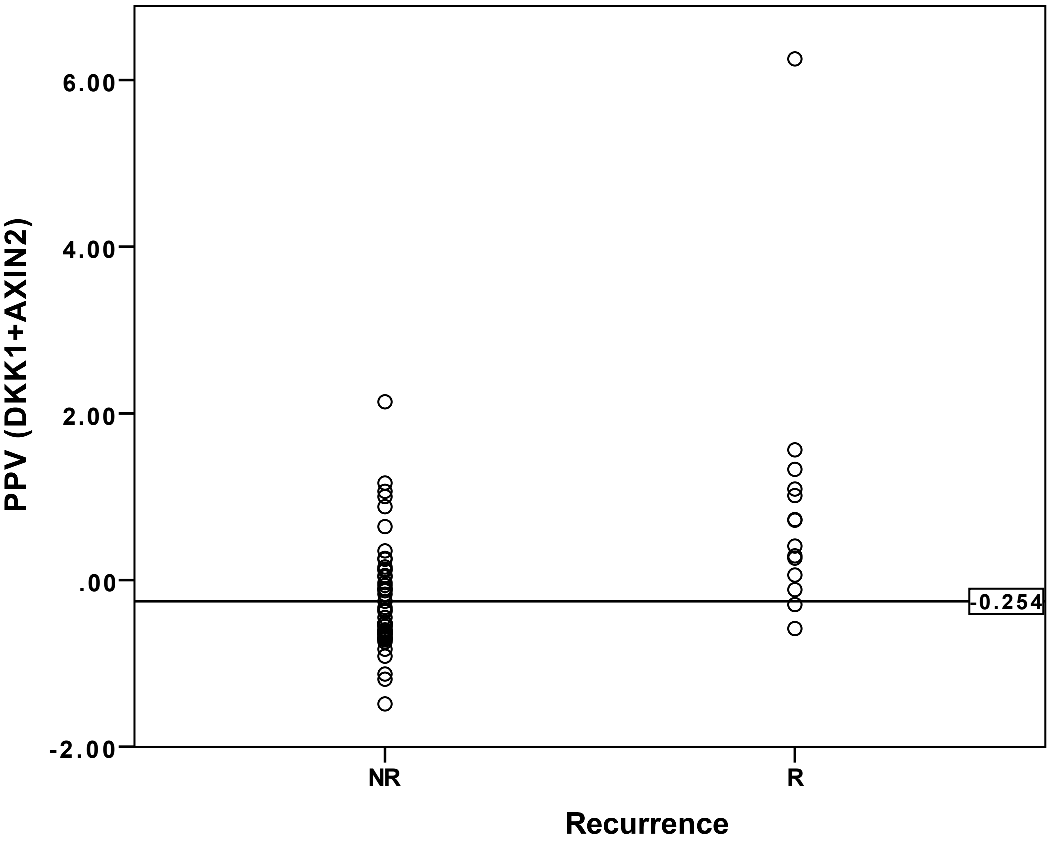

B

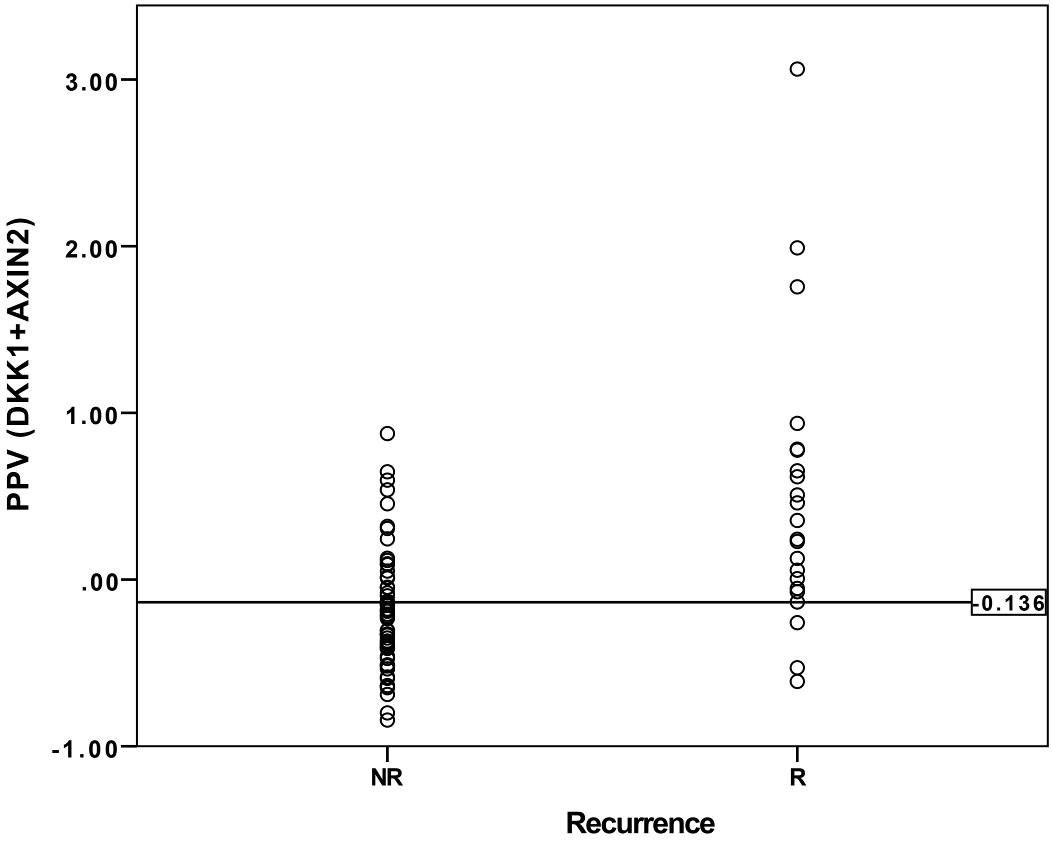

Supplement: Supplementary Figure 1 [file oncsis20179x3.pdf]
